# Supplementary material for: A Multicriteria Approach to Find Predictive and Sparse Models with Stable Feature Selection for High-Dimensional Data
Source: Comput Math Methods Med. 2017 Aug 1;2017:7907163. doi: 10.1155/2017/7907163 (PMC5556617; doi:10.1155/2017/7907163)
Supplement: Supplementary file 1 — Results of the study for finding desirable configurations for all stability measures. [file 7907163.f1.pdf]

Supplementary Material for “A Multicriteria Approach to Find Predictive and Sparse Models with Stable Feature Selection for High-Dimensional Data”

Andrea Bommert, Jörg Rahnenführer, and Michel Lang

Results of the Study for Finding Desirable Configurations for All Stability Measures

AP_Breast_Ovary 2

AP_Colon_Kidney 6

Stomach 12

AP_Breast_Ovary

ID	Classifier	Parameters	Filter	`n.feats`	Error (train)	Error (test)	Size (train)	Size (test)
1	Lasso. Log. Reg.	$\lambda = 0.0002$	Variance	10933	0.037	0.056	10932.8	10932.4
2	Lasso. Log. Reg.	$\lambda = 0.001$	Variance	10865	0.026	0.048	10864.2	10863.6
3	Lasso. Log. Reg.	$\lambda = 0.001$	Variance	10725	0.045	0.026	10724.0	10724.0
4	Lasso. Log. Reg.	$\lambda = 0.0005$	Variance	10622	0.033	0.044	10621.8	10621.7
5	Lasso. Log. Reg.	$\lambda = 0.0001$	Variance	10543	0.037	0.040	10542.9	10542.7
6	Lasso. Log. Reg.	$\lambda = 0.0001$	Variance	10509	0.019	0.040	10508.9	10508.9
7	Lasso. Log. Reg.	$\lambda = 0.003$	Variance	10488	0.026	0.052	10484.6	10485.0
8	Lasso. Log. Reg.	$\lambda = 0.0001$	Variance	10477	0.033	0.044	10476.9	10476.9
9	Lasso. Log. Reg.	$\lambda = 0.003$	Variance	10448	0.044	0.037	10445.2	10445.2
10	Random Forest	`num.trees` = 18741, `min.node.size` = 1	Variance	10447	0.040	0.022	10403.8	10407.5
11	Lasso. Log. Reg.	$\lambda = 0.0001$	Variance	10313	0.030	0.026	10312.7	10312.7
12	Lasso. Log. Reg.	$\lambda = 0.001$	Variance	10256	0.022	0.048	10254.7	10255.8
13	Lasso. Log. Reg.	$\lambda = 0.014$	Variance	10196	0.030	0.052	10183.8	10185.4
14	Lasso. Log. Reg.	$\lambda = 0.004$	Variance	10044	0.033	0.037	10040.6	10041.0
15	Lasso. Log. Reg.	$\lambda = 0.00005$	Variance	10030	0.044	0.030	10030.0	10029.9
16	Lasso. Log. Reg.	$\lambda = 0.0005$	Variance	9995	0.033	0.033	9994.6	9994.7
17	Lasso. Log. Reg.	$\lambda = 0.019$	Variance	9926	0.019	0.055	9910.4	9910.7
18	Lasso. Log. Reg.	$\lambda = 0.025$	Variance	9927	0.023	0.030	9906.2	9905.8
19	Lasso. Log. Reg.	$\lambda = 0.003$	Variance	9523	0.022	0.022	9521.0	9520.8
20	Lasso. Log. Reg.	$\lambda = 0.0002$	Variance	9373	0.026	0.052	9373.0	9372.8
21	Lasso. Log. Reg.	$\lambda = 0.00004$	Variance	9239	0.022	0.041	9239.0	9239.0
22	Lasso. Log. Reg.	$\lambda = 0.0003$	Variance	8945	0.029	0.030	8944.9	8944.9
23	SVM	$C = 4424.956$, $\sigma = 0.00003$	Variance	8939	0.044	0.026	8939.0	8939.0
24	Lasso. Log. Reg.	$\lambda = 0.0004$	Variance	8843	0.037	0.041	8843.0	8843.0
25	Lasso. Log. Reg.	$\lambda = 0.0001$	Variance	8837	0.040	0.026	8837.0	8836.9
26	Lasso. Log. Reg.	$\lambda = 0.005$	Variance	8615	0.022	0.051	8612.9	8612.5
27	Lasso. Log. Reg.	$\lambda = 0.01$	Variance	8198	0.037	0.041	8193.8	8192.8
28	Lasso. Log. Reg.	$\lambda = 0.0002$	Variance	7767	0.040	0.019	7767.0	7766.7
29	Lasso. Log. Reg.	$\lambda = 0.001$	Variance	7042	0.048	0.037	7041.5	7041.9
30	Random Forest	`num.trees` = 24717, `min.node.size` = 2	Variance	6522	0.036	0.026	6521.7	6521.7
31	Lasso. Log. Reg.	$\lambda = 0.0001$	Variance	5371	0.033	0.040	5371.0	5371.0
32	Lasso. Log. Reg.	$\lambda = 0.002$	Variance	4363	0.026	0.048	4362.8	4362.8
33	Lasso. Log. Reg.	$\lambda = 0.00005$	Variance	420	0.041	0.041	420.0	420.0
34	Random Forest	`num.trees` = 20577, `min.node.size` = 2	Variance	397	0.041	0.048	397.0	397.0
35	SVM	$C = 81.668$, $\sigma = 0.0001$	Variance	355	0.040	0.044	355.0	355.0
36	Lasso. Log. Reg.	$\lambda = 51.202$	Variance	349	0.048	0.030	347.5	346.9
37	Lasso. Log. Reg.	$\lambda = 2.06$	Variance	246	0.041	0.059	246.0	245.9
38	Lasso. Log. Reg.	$\lambda = 0.133$	Variance	232	0.037	0.044	232.0	232.0
39	Random Forest	`num.trees` = 4742, `min.node.size` = 2	Variance	212	0.048	0.044	212.0	212.0
40	Lasso. Log. Reg.	$\lambda = 0.085$	Variance	174	0.044	0.048	174.0	174.0
41	GLM Boosting	$m_{\text{stop}} = 27$	Variance	728	0.041	0.041	13.3	15.1
42	GLM Boosting	$m_{\text{stop}} = 25$	Variance	363	0.048	0.074	9.8	7.2
43	GLM Boosting	$m_{\text{stop}} = 13$	AUC	232	0.048	0.077	9.7	11.0
44	Random Forest	`num.trees` = 1062, `min.node.size` = 3	MRMR	1756	0.000	0.055	1466.0	1611.9
45	Random Forest	`num.trees` = 4671, `min.node.size` = 11	AUC	760	0.000	0.051	759.7	760.0

Table A: All Pareto optimal configurations for data set AP_Breast_Ovary (above the horizontal line) and accuracy optimal configurations (below the horizontal line). The ID column references Tables B, C, and D.

ID	1 - SJ (train)	1 - SJ (test)	1 - SC (train)	1 - SC (test)	1 - SD-1 (train)	1 - SD-1 (test)	1 - SD-10 (train)	1 - SD-10 (test)
1	0.000	0.000	—	—	—	—	—	—
2	0.002	0.003	—	—	—	—	—	—
3	0.010	0.007	—	—	—	—	—	—
4	0.011	0.014	—	—	—	—	—	—
5	0.015	0.017	—	—	—	—	—	—
6	0.015	0.019	—	—	—	—	—	—
7	0.017	0.018	—	—	—	—	—	—
8	0.018	0.018	—	—	—	—	—	—
9	0.023	0.018	—	—	—	—	—	—
10	—	—	—	—	—	—	—	—
11	—	—	—	—	—	—	—	—
12	0.023	0.028	—	—	—	—	—	—
13	0.029	0.032	—	—	—	—	—	—
14	0.031	0.032	—	—	—	—	—	—
15	0.031	0.032	—	—	—	—	—	—
16	0.032	0.032	—	—	—	—	—	—
17	0.034	0.038	—	—	—	—	—	—
18	—	—	—	—	—	—	—	—
19	0.035	0.039	—	—	—	—	—	—
20	0.037	0.038	—	—	—	—	—	—
21	—	—	—	—	—	—	—	—
22	0.038	0.040	—	—	—	—	—	—
23	0.038	0.040	—	—	—	—	—	—
24	0.038	0.040	—	—	—	—	—	—
25	—	—	—	—	—	—	—	—
26	—	—	—	—	—	—	—	—
27	—	—	—	—	—	—	—	—
28	—	—	—	—	—	—	—	—
29	—	—	—	—	—	—	—	—
30	—	—	—	—	—	—	—	—
31	—	—	—	—	—	—	—	—
32	—	—	—	—	—	—	—	—
33	—	—	—	—	—	—	—	—
34	0.039	0.046	0.021	0.025	—	—	—	—
35	0.040	0.054	0.021	0.029	—	—	—	—
36	—	—	—	—	—	—	—	—
37	0.041	0.060	0.021	0.032	0.062	0.091	—	—
38	0.047	0.060	0.025	0.032	0.066	0.097	—	—
39	0.063	0.054	0.034	0.028	0.081	0.073	—	—
40	0.072	0.057	0.038	0.030	0.100	0.090	0.243	0.234
41	0.178	0.340	0.100	0.208	—	—	—	—
42	0.206	0.283	0.117	0.169	0.247	0.447	0.255	0.453
43	0.482	0.503	0.325	0.341	0.597	0.561	0.605	0.570
44	0.345	0.284	0.240	0.195	0.455	0.441	1.000	1.000
45	0.131	0.158	0.075	0.092	0.253	0.277	0.879	0.903

Table B: Stability values for the Pareto optimal configurations in Table A. The ID column references Table A. "—" means that the configuration is not Pareto optimal for the corresponding stability measure.

ID	1 - SD-0 (train)	1 - SD-0 (test)	1 - SD-2 (train)	1 - SD-2 (test)	1 - SD (train)	1 - SD (test)	1 - SL (train)	1 - SL (test)
1	0.000	0.000	—	—	0.000	0.000	0.008	0.771
2	0.001	0.002	—	—	0.001	0.001	—	—
3	0.006	0.005	—	—	0.005	0.004	—	—
4	0.007	0.010	—	—	0.005	0.007	—	—
5	0.010	0.012	—	—	0.007	0.009	—	—
6	0.010	0.013	—	—	0.007	0.010	—	—
7	0.011	0.013	—	—	0.008	0.009	—	—
8	0.012	0.012	—	—	0.009	0.009	—	—
9	0.015	0.013	—	—	0.011	0.009	—	—
10	0.016	0.019	—	—	—	—	—	—
11	0.017	0.019	—	—	—	—	—	—
12	0.018	0.021	—	—	0.012	0.014	—	—
13	0.021	0.023	—	—	0.015	0.016	—	—
14	—	—	—	—	0.016	0.016	—	—
15	0.023	0.025	—	—	0.016	0.016	—	—
16	0.025	0.025	—	—	0.016	0.016	—	—
17	0.026	0.029	—	—	0.017	0.019	—	—
18	0.027	0.026	—	—	—	—	—	—
19	0.028	0.031	—	—	0.018	0.020	—	—
20	—	—	—	—	0.019	0.020	—	—
21	0.029	0.031	—	—	—	—	—	—
22	0.030	0.032	—	—	0.019	0.020	—	—
23	0.030	0.031	—	—	0.019	0.020	—	—
24	—	—	—	—	0.020	0.021	—	—
25	0.031	0.031	—	—	—	—	—	—
26	0.031	0.032	—	—	—	—	—	—
27	0.033	0.035	—	—	—	—	—	—
28	0.033	0.033	—	—	—	—	—	—
29	0.033	0.036	—	—	—	—	—	—
30	0.035	0.036	—	—	—	—	—	—
31	0.035	0.040	—	—	—	—	—	—
32	0.036	0.036	—	—	—	—	—	—
33	0.037	0.043	—	—	—	—	—	—
34	—	—	—	—	0.020	0.024	—	—
35	—	—	—	—	0.020	0.028	—	—
36	0.037	0.060	—	—	—	—	—	—
37	0.039	0.069	0.084	0.114	0.021	0.031	0.043	0.054
38	0.045	0.076	0.088	0.118	0.024	0.031	0.046	0.053
39	0.062	0.054	0.101	0.092	0.033	0.028	0.052	0.047
40	0.084	0.074	0.116	0.106	0.038	0.029	0.053	0.045
41	—	—	—	—	0.101	0.209	0.069	0.179
42	0.246	0.446	0.248	0.447	0.118	0.170	0.077	0.130
43	0.596	0.560	0.598	0.562	0.325	0.342	0.298	0.313
44	0.321	0.294	0.589	0.589	0.208	0.166	0.337	0.309
45	0.184	0.208	0.323	0.347	0.070	0.086	0.139	0.155

Table C: Stability values for the Pareto optimal configurations in Table A. The ID column references Table A. "—" means that the configuration is not Pareto optimal for the corresponding stability measure.

ID	1 - SN (train)	1 - SN (test)	1 - SO (train)	1 - SO (test)	1 - SS (train)	1 - SS (test)	1 - SZ (train)	1 - SZ (test)
1	0.000	0.000	0.000	0.000	0.011	0.800	0.000	0.000
2	0.000	0.001	0.001	0.001	—	—	0.002	0.003
3	0.002	0.002	0.005	0.004	—	—	0.009	0.007
4	0.002	0.003	0.005	0.007	—	—	0.010	0.014
5	0.003	0.004	0.007	0.009	—	—	0.014	0.016
6	0.003	0.004	0.007	0.010	—	—	0.014	0.018
7	0.004	0.004	0.008	0.009	—	—	0.016	0.017
8	0.004	0.004	0.009	0.009	—	—	0.017	0.018
9	0.005	0.004	0.011	0.009	—	—	0.021	0.017
10	—	—	—	—	—	—	—	—
11	0.006	0.006	—	—	—	—	—	—
12	0.006	0.007	0.012	0.014	—	—	0.022	0.026
13	0.007	0.008	0.015	0.016	—	—	0.027	0.030
14	—	—	0.016	0.016	—	—	0.029	0.030
15	0.007	0.008	0.016	0.016	—	—	0.029	0.030
16	0.008	0.008	0.016	0.016	—	—	0.031	0.030
17	0.008	0.009	0.017	0.019	—	—	0.032	0.036
18	—	—	—	—	—	—	—	—
19	0.009	0.010	0.018	0.020	—	—	0.033	0.037
20	0.009	0.010	0.019	0.020	—	—	—	—
21	—	—	—	—	—	—	—	—
22	0.009	0.010	0.019	0.020	—	—	—	—
23	0.009	0.010	0.019	0.020	—	—	—	—
24	0.010	0.010	0.020	0.021	—	—	—	—
25	0.010	0.010	—	—	—	—	—	—
26	0.010	0.010	—	—	—	—	—	—
27	0.010	0.011	—	—	—	—	—	—
28	—	—	—	—	—	—	—	—
29	—	—	—	—	—	—	—	—
30	—	—	—	—	—	—	—	—
31	—	—	—	—	—	—	—	—
32	—	—	—	—	—	—	—	—
33	—	—	—	—	—	—	—	—
34	0.010	0.013	0.020	0.024	0.020	0.024	—	—
35	—	—	0.020	0.028	0.020	0.028	—	—
36	—	—	—	—	—	—	—	—
37	0.011	0.017	0.021	0.031	0.021	0.031	0.034	0.052
38	0.013	0.018	0.024	0.031	0.024	0.031	0.039	0.050
39	0.017	0.015	0.033	0.028	0.033	0.028	0.050	0.046
40	0.021	0.017	0.038	0.029	0.038	0.029	0.056	0.044
41	0.066	0.129	0.100	0.208	0.085	0.204	0.145	0.281
42	0.068	0.125	0.117	0.169	0.102	0.149	0.175	0.249
43	0.233	0.234	0.324	0.341	0.308	0.343	0.387	0.382
44	0.116	0.095	0.208	0.166	0.221	0.179	0.324	0.268
45	0.044	0.053	0.070	0.086	0.070	0.086	0.118	0.142

Table D: Stability values for the Pareto optimal configurations in Table A. The ID column references Table A. "—" means that the configuration is not Pareto optimal for the corresponding stability measure.

AP_Colon_Kidney

ID	Classifier	Parameters	Filter	n.feats	Error (train)	Error (test)	Size (train)	Size (test)
46	GLM Boosting	$m_{stop} = 1$	AUC	7850	0.044	0.063	1.0	1.0
47	GLM Boosting	$m_{stop} = 1$	AUC	6132	0.026	0.041	1.0	1.0
48	GLM Boosting	$m_{stop} = 1$	AUC	5010	0.029	0.059	1.0	1.0
49	GLM Boosting	$m_{stop} = 1$	AUC	10398	0.036	0.066	1.0	1.0
50	GLM Boosting	$m_{stop} = 1$	AUC	4785	0.029	0.069	1.0	1.0
51	GLM Boosting	$m_{stop} = 1$	AUC	4796	0.044	0.040	1.0	1.0
52	GLM Boosting	$m_{stop} = 1$	AUC	6976	0.036	0.048	1.0	1.0
53	GLM Boosting	$m_{stop} = 1$	AUC	1419	0.018	0.055	1.0	1.0
54	GLM Boosting	$m_{stop} = 1$	AUC	150	0.037	0.051	1.0	1.0
55	GLM Boosting	$m_{stop} = 1$	AUC	4540	0.033	0.051	1.0	1.0
56	GLM Boosting	$m_{stop} = 1$	AUC	2917	0.029	0.047	1.0	1.0
57	GLM Boosting	$m_{stop} = 1$	AUC	2137	0.011	0.143	1.0	1.0
58	GLM Boosting	$m_{stop} = 1$	AUC	1136	0.026	0.040	1.0	1.0
59	GLM Boosting	$m_{stop} = 1$	AUC	5517	0.022	0.139	1.0	1.0
60	GLM Boosting	$m_{stop} = 2$	AUC	8921	0.029	0.044	1.0	1.1
61	GLM Boosting	$m_{stop} = 1$	AUC	5860	0.033	0.077	1.0	1.0
62	GLM Boosting	$m_{stop} = 1$	AUC	7749	0.037	0.048	1.0	1.0
63	GLM Boosting	$m_{stop} = 1$	AUC	9962	0.022	0.055	1.0	1.0
64	GLM Boosting	$m_{stop} = 1$	AUC	1520	0.040	0.062	1.0	1.0
65	GLM Boosting	$m_{stop} = 1$	AUC	5067	0.036	0.047	1.0	1.0
66	GLM Boosting	$m_{stop} = 1$	AUC	10106	0.040	0.029	1.0	1.0
67	GLM Boosting	$m_{stop} = 1$	AUC	8684	0.037	0.044	1.0	1.0
68	GLM Boosting	$m_{stop} = 1$	AUC	2535	0.037	0.074	1.0	1.0
69	GLM Boosting	$m_{stop} = 1$	AUC	6221	0.037	0.040	1.0	1.0
70	GLM Boosting	$m_{stop} = 1$	MRMR	5790	0.040	0.041	1.0	1.0
71	GLM Boosting	$m_{stop} = 1$	MRMR	6915	0.044	0.033	1.0	1.0
72	GLM Boosting	$m_{stop} = 1$	MRMR	7647	0.041	0.037	1.0	1.0
73	GLM Boosting	$m_{stop} = 1$	MRMR	5301	0.015	0.088	1.0	1.0
74	GLM Boosting	$m_{stop} = 1$	MRMR	7718	0.029	0.066	1.0	1.0
75	GLM Boosting	$m_{stop} = 1$	MRMR	7339	0.041	0.048	1.0	1.0
76	GLM Boosting	$m_{stop} = 1$	MRMR	10219	0.026	0.143	1.0	1.0
77	GLM Boosting	$m_{stop} = 1$	MRMR	7006	0.033	0.069	1.0	1.0
78	GLM Boosting	$m_{stop} = 1$	MRMR	4521	0.048	0.129	1.0	1.0
79	GLM Boosting	$m_{stop} = 1$	MRMR	8702	0.029	0.044	1.0	1.0
80	GLM Boosting	$m_{stop} = 1$	MRMR	475	0.037	0.150	1.0	1.0
81	GLM Boosting	$m_{stop} = 1$	MRMR	9426	0.033	0.058	1.0	1.0
82	GLM Boosting	$m_{stop} = 1$	MRMR	9081	0.029	0.041	1.0	1.0
83	GLM Boosting	$m_{stop} = 1$	MRMR	4783	0.029	0.110	1.0	1.0
84	GLM Boosting	$m_{stop} = 1$	Variance	5344	0.040	0.051	1.0	1.0
85	GLM Boosting	$m_{stop} = 1$	Variance	1702	0.029	0.033	1.0	1.0
86	GLM Boosting	$m_{stop} = 1$	Variance	10831	0.040	0.026	1.0	1.0
87	GLM Boosting	$m_{stop} = 1$	Variance	6173	0.047	0.048	1.0	1.0
88	GLM Boosting	$m_{stop} = 1$	Variance	1717	0.029	0.069	1.0	1.0
89	GLM Boosting	$m_{stop} = 1$	Variance	1578	0.040	0.051	1.0	1.0
90	GLM Boosting	$m_{stop} = 1$	Variance	3102	0.026	0.088	1.0	1.0
91	GLM Boosting	$m_{stop} = 1$	Variance	632	0.048	0.070	1.0	1.0
92	GLM Boosting	$m_{stop} = 1$	Variance	2895	0.037	0.055	1.0	1.0
93	GLM Boosting	$m_{stop} = 1$	Variance	5111	0.037	0.051	1.0	1.0
94	GLM Boosting	$m_{stop} = 1$	Variance	2279	0.029	0.069	1.0	1.0
95	GLM Boosting	$m_{stop} = 1$	Variance	1638	0.041	0.048	1.0	1.0
96	GLM Boosting	$m_{stop} = 1$	Variance	2818	0.036	0.044	1.0	1.0
97	GLM Boosting	$m_{stop} = 1$	Variance	4960	0.048	0.029	1.0	1.0
98	GLM Boosting	$m_{stop} = 1$	Variance	9519	0.033	0.040	1.0	1.0
99	GLM Boosting	$m_{stop} = 1$	Variance	8416	0.033	0.055	1.0	1.0
100	GLM Boosting	$m_{stop} = 1$	Variance	2235	0.037	0.033	1.0	1.0
101	GLM Boosting	$m_{stop} = 1$	Variance	7155	0.037	0.077	1.0	1.0
102	GLM Boosting	$m_{stop} = 1$	Variance	10093	0.033	0.058	1.0	1.0

ID	Classifier	Parameters	Filter	n.feats	Error (train)	Error (test)	Size (train)	Size (test)
103	GLM Boosting	$m_{\text{stop}} = 1$	Variance	5376	0.033	0.058	1.0	1.0
104	GLM Boosting	$m_{\text{stop}} = 1$	Variance	8198	0.026	0.062	1.0	1.0
105	GLM Boosting	$m_{\text{stop}} = 1$	Variance	5870	0.029	0.051	1.0	1.0
106	GLM Boosting	$m_{\text{stop}} = 1$	Variance	2274	0.033	0.077	1.0	1.0
107	GLM Boosting	$m_{\text{stop}} = 1$	Variance	3881	0.033	0.047	1.0	1.0
108	GLM Boosting	$m_{\text{stop}} = 1$	Variance	189	0.040	0.077	1.0	1.0
109	GLM Boosting	$m_{\text{stop}} = 1$	Variance	3235	0.036	0.047	1.0	1.0
110	GLM Boosting	$m_{\text{stop}} = 1$	Variance	241	0.048	0.069	1.0	1.0
111	GLM Boosting	$m_{\text{stop}} = 1$	Variance	3490	0.037	0.033	1.0	1.0
112	GLM Boosting	$m_{\text{stop}} = 2$	Variance	3765	0.033	0.029	1.0	2.0
113	Lasso. Log. Reg.	$\lambda = 0.0001$	Variance	10812	0.000	0.026	10811.9	10812.0
114	Lasso. Log. Reg.	$\lambda = 20.559$	MRMR	9927	0.000	0.025	4546.2	4728.2
115	GLM Boosting	$m_{\text{stop}} = 28692$	Variance	972	0.000	0.026	51.0	55.4
116	GLM Boosting	$m_{\text{stop}} = 213$	Variance	1586	0.000	0.040	25.7	42.5

Table E: All Pareto optimal configurations for data set AP_Colon_Kidney (above the horizontal line) and accuracy optimal configurations (below the horizontal line). The ID column references Tables F, G, and H.

ID	1 - SJ (train)	1 - SJ (test)	1 - SC (train)	1 - SC (test)	1 - SD-1 (train)	1 - SD-1 (test)	1 - SD-10 (train)	1 - SD-10 (test)
46	0.000	0.467	0.000	0.467	0.000	0.500	0.001	0.501
47	0.000	0.000	0.000	0.000	0.000	0.000	0.001	0.001
48	0.000	0.200	0.000	0.200	0.000	0.500	0.001	0.501
49	0.000	0.356	0.000	0.356	0.000	0.500	0.001	0.501
50	0.000	0.511	0.000	0.511	0.000	0.667	0.001	0.668
51	0.000	0.200	0.000	0.200	0.000	0.500	0.001	0.501
52	0.000	0.467	0.000	0.467	0.000	0.500	0.001	0.501
53	0.000	0.200	0.000	0.200	0.000	0.500	0.001	0.501
54	0.000	0.200	0.000	0.200	0.000	0.500	0.001	0.501
55	0.000	0.200	0.000	0.200	0.000	0.500	0.001	0.501
56	0.000	0.000	0.000	0.000	0.000	0.000	0.001	0.001
57	0.000	0.511	0.000	0.511	0.000	0.667	0.001	0.668
58	0.000	0.000	0.000	0.000	0.000	0.000	0.001	0.001
59	0.000	0.000	0.000	0.000	0.000	0.000	0.001	0.001
60	0.000	0.100	0.000	0.059	0.000	0.450	0.001	0.451
61	0.000	0.622	0.000	0.622	0.000	0.667	0.001	0.668
62	0.000	0.356	0.000	0.356	0.000	0.500	0.001	0.501
63	0.000	0.533	0.000	0.533	0.000	0.500	0.001	0.501
64	0.000	0.556	0.000	0.556	0.000	0.500	0.001	0.501
65	0.000	0.000	0.000	0.000	0.000	0.000	0.001	0.001
66	0.000	0.000	0.000	0.000	0.000	0.000	0.001	0.001
67	0.000	0.200	0.000	0.200	0.000	0.500	0.001	0.501
68	0.000	0.511	0.000	0.511	0.000	0.667	0.001	0.668
69	0.000	0.356	0.000	0.356	0.000	0.500	0.001	0.501
70	0.000	0.200	0.000	0.200	0.000	0.500	0.001	0.501
71	0.000	0.689	0.000	0.689	0.000	0.667	0.001	0.668
72	0.000	0.356	0.000	0.356	0.000	0.500	0.001	0.501
73	0.000	0.356	0.000	0.356	0.000	0.500	0.001	0.501
74	0.000	0.356	0.000	0.356	0.000	0.500	0.001	0.501
75	0.000	0.356	0.000	0.356	0.000	0.500	0.001	0.501
76	0.000	0.378	0.000	0.378	0.000	0.667	0.001	0.668
77	0.000	0.644	0.000	0.645	0.000	0.667	0.001	0.668
78	0.000	0.644	0.000	0.645	0.000	0.750	0.001	0.751
79	0.000	0.000	0.000	0.000	0.000	0.000	0.001	0.001
80	0.000	0.511	0.000	0.511	0.000	0.667	0.001	0.668
81	0.000	0.467	0.000	0.467	0.000	0.500	0.001	0.501
82	0.000	0.533	0.000	0.533	0.000	0.500	0.001	0.501
83	0.000	0.533	0.000	0.533	0.000	0.500	0.001	0.501
84	0.000	0.200	0.000	0.200	0.000	0.500	0.001	0.501

ID	1 - SJ (train)	1 - SJ (test)	1 - SC (train)	1 - SC (test)	1 - SD-1 (train)	1 - SD-1 (test)	1 - SD-10 (train)	1 - SD-10 (test)
85	0.000	0.000	0.000	0.000	0.000	0.000	0.001	0.001
86	0.000	0.000	0.000	0.000	0.000	0.000	0.001	0.001
87	0.000	0.000	0.000	0.000	0.000	0.000	0.001	0.001
88	0.000	0.467	0.000	0.467	0.000	0.500	0.001	0.501
89	0.000	0.356	0.000	0.356	0.000	0.500	0.001	0.501
90	0.000	0.600	0.000	0.600	0.000	0.667	0.001	0.668
91	0.000	0.000	0.000	0.000	0.000	0.000	0.001	0.001
92	0.000	0.000	0.000	0.000	0.000	0.000	0.001	0.001
93	0.000	0.200	0.000	0.200	0.000	0.500	0.001	0.501
94	0.000	0.533	0.000	0.533	0.000	0.500	0.001	0.501
95	0.000	0.356	0.000	0.356	0.000	0.500	0.001	0.501
96	0.000	0.533	0.000	0.533	0.000	0.500	0.001	0.501
97	0.000	0.000	0.000	0.000	0.000	0.000	0.001	0.001
98	0.000	0.000	0.000	0.000	0.000	0.000	0.001	0.001
99	0.000	0.200	0.000	0.200	0.000	0.500	0.001	0.501
100	0.000	0.000	0.000	0.000	0.000	0.000	0.001	0.001
101	0.000	0.622	0.000	0.622	0.000	0.667	0.001	0.668
102	0.000	0.556	0.000	0.556	0.000	0.500	0.001	0.501
103	0.000	0.467	0.000	0.467	0.000	0.500	0.001	0.501
104	0.000	0.200	0.000	0.200	0.000	0.500	0.001	0.501
105	0.000	0.200	0.000	0.200	0.000	0.500	0.001	0.501
106	0.000	0.622	0.000	0.622	0.000	0.667	0.001	0.668
107	0.000	0.000	0.000	0.000	0.000	0.000	0.001	0.001
108	0.000	0.000	0.000	0.000	0.000	0.000	0.001	0.001
109	0.000	0.000	0.000	0.000	0.000	0.000	0.001	0.001
110	0.000	0.000	0.000	0.000	0.000	0.000	0.001	0.001
111	0.000	0.000	0.000	0.000	0.000	0.000	0.001	0.001
112	0.000	0.000	0.000	0.000	0.000	0.000	0.001	0.002
113	0.004	0.004	0.188	0.168	0.993	0.991	1.000	1.000
114	0.563	0.557	0.671	0.680	0.934	0.944	1.000	1.000
115	0.426	0.553	0.274	0.387	0.468	0.601	0.510	0.646
116	0.440	0.519	0.286	0.355	0.543	0.566	0.565	0.602

Table F: Stability values for the Pareto optimal configurations in Table E. The ID column references Table E. "—" means that the configuration is not Pareto optimal for the corresponding stability measure.

ID	1 - SD-0 (train)	1 - SD-0 (test)	1 - SD-2 (train)	1 - SD-2 (test)	1 - SD (train)	1 - SD (test)	1 - SL (train)	1 - SL (test)
46	0.000	0.500	0.000	0.500	0.000	0.467	0.000	0.467
47	0.000	0.000	0.000	0.000	0.000	0.000	0.000	0.000
48	0.000	0.500	0.000	0.500	0.000	0.200	0.000	0.200
49	0.000	0.500	0.000	0.500	0.000	0.356	0.000	0.356
50	0.000	0.667	0.000	0.667	0.000	0.511	0.000	0.511
51	0.000	0.500	0.000	0.500	0.000	0.200	0.000	0.200
52	0.000	0.500	0.000	0.500	0.000	0.467	0.000	0.467
53	0.000	0.500	0.000	0.500	0.000	0.200	0.000	0.200
54	0.000	0.500	0.000	0.500	0.000	0.200	0.000	0.200
55	0.000	0.500	0.000	0.500	0.000	0.200	0.000	0.200
56	0.000	0.000	0.000	0.000	0.000	0.000	0.000	0.000
57	0.000	0.667	0.000	0.667	0.000	0.511	0.000	0.511
58	0.000	0.000	0.000	0.000	0.000	0.000	0.000	0.000
59	0.000	0.000	0.000	0.000	0.000	0.000	0.000	0.000
60	0.000	0.450	0.000	0.450	0.000	0.067	0.000	0.000
61	0.000	0.667	0.000	0.667	0.000	0.622	0.000	0.622
62	0.000	0.500	0.000	0.500	0.000	0.356	0.000	0.356
63	0.000	0.500	0.000	0.500	0.000	0.533	0.000	0.533
64	0.000	0.500	0.000	0.500	0.000	0.556	0.000	0.556
65	0.000	0.000	0.000	0.000	0.000	0.000	0.000	0.000
66	0.000	0.000	0.000	0.000	0.000	0.000	0.000	0.000

ID	1 - SD-0 (train)	1 - SD-0 (test)	1 - SD-2 (train)	1 - SD-2 (test)	1 - SD (train)	1 - SD (test)	1 - SL (train)	1 - SL (test)
67	0.000	0.500	0.000	0.500	0.000	0.200	0.000	0.200
68	0.000	0.667	0.000	0.667	0.000	0.511	0.000	0.511
69	0.000	0.500	0.000	0.500	0.000	0.356	0.000	0.356
70	0.000	0.500	0.000	0.500	0.000	0.200	0.000	0.200
71	0.000	0.667	0.000	0.667	0.000	0.689	0.000	0.689
72	0.000	0.500	0.000	0.500	0.000	0.356	0.000	0.356
73	0.000	0.500	0.000	0.500	0.000	0.356	0.000	0.356
74	0.000	0.500	0.000	0.500	0.000	0.356	0.000	0.356
75	0.000	0.500	0.000	0.500	0.000	0.356	0.000	0.356
76	0.000	0.667	0.000	0.667	0.000	0.378	0.000	0.378
77	0.000	0.667	0.000	0.667	0.000	0.644	0.000	0.645
78	0.000	0.750	0.000	0.750	0.000	0.644	0.000	0.645
79	0.000	0.000	0.000	0.000	0.000	0.000	0.000	0.000
80	0.000	0.667	0.000	0.667	0.000	0.511	0.000	0.511
81	0.000	0.500	0.000	0.500	0.000	0.467	0.000	0.467
82	0.000	0.500	0.000	0.500	0.000	0.533	0.000	0.533
83	0.000	0.500	0.000	0.500	0.000	0.533	0.000	0.533
84	0.000	0.500	0.000	0.500	0.000	0.200	0.000	0.200
85	0.000	0.000	0.000	0.000	0.000	0.000	0.000	0.000
86	0.000	0.000	0.000	0.000	0.000	0.000	0.000	0.000
87	0.000	0.000	0.000	0.000	0.000	0.000	0.000	0.000
88	0.000	0.500	0.000	0.500	0.000	0.467	0.000	0.467
89	0.000	0.500	0.000	0.500	0.000	0.356	0.000	0.356
90	0.000	0.667	0.000	0.667	0.000	0.600	0.000	0.600
91	0.000	0.000	0.000	0.000	0.000	0.000	0.000	0.000
92	0.000	0.000	0.000	0.000	0.000	0.000	0.000	0.000
93	0.000	0.500	0.000	0.500	0.000	0.200	0.000	0.200
94	0.000	0.500	0.000	0.500	0.000	0.533	0.000	0.533
95	0.000	0.500	0.000	0.500	0.000	0.356	0.000	0.356
96	0.000	0.500	0.000	0.500	0.000	0.533	0.000	0.533
97	0.000	0.000	0.000	0.000	0.000	0.000	0.000	0.000
98	0.000	0.000	0.000	0.000	0.000	0.000	0.000	0.000
99	0.000	0.500	0.000	0.500	0.000	0.200	0.000	0.200
100	0.000	0.000	0.000	0.000	0.000	0.000	0.000	0.000
101	0.000	0.667	0.000	0.667	0.000	0.622	0.000	0.622
102	0.000	0.500	0.000	0.500	0.000	0.556	0.000	0.556
103	0.000	0.500	0.000	0.500	0.000	0.467	0.000	0.467
104	0.000	0.500	0.000	0.500	0.000	0.200	0.000	0.200
105	0.000	0.500	0.000	0.500	0.000	0.200	0.000	0.200
106	0.000	0.667	0.000	0.667	0.000	0.622	0.000	0.622
107	0.000	0.000	0.000	0.000	0.000	0.000	0.000	0.000
108	0.000	0.000	0.000	0.000	0.000	0.000	0.000	0.000
109	0.000	0.000	0.000	0.000	0.000	0.000	0.000	0.000
110	0.000	0.000	0.000	0.000	0.000	0.000	0.000	0.000
111	0.000	0.000	0.000	0.000	0.000	0.000	0.000	0.000
112	0.000	0.000	0.000	0.000	0.000	0.000	0.000	0.000
113	0.004	0.003	1.000	1.000	0.002	0.002	0.196	0.177
114	0.520	0.508	1.000	1.000	0.392	0.386	0.805	0.816
115	0.463	0.596	0.472	0.606	0.274	0.387	0.242	0.352
116	0.541	0.562	0.546	0.570	0.287	0.355	0.250	0.321

Table G: Stability values for the Pareto optimal configurations in Table E. The ID column references Table E. "—" means that the configuration is not Pareto optimal for the corresponding stability measure.

ID	1 - SN (train)	1 - SN (test)	1 - SO (train)	1 - SO (test)	1 - SS (train)	1 - SS (test)	1 - SZ (train)	1 - SZ (test)
46	0.000	0.265	0.000	0.467	0.000	0.467	0.000	0.467
47	0.000	0.000	0.000	0.000	0.000	0.000	0.000	0.000
48	0.000	0.141	0.000	0.200	0.000	0.200	0.000	0.200
49	0.000	0.217	0.000	0.356	0.000	0.356	0.000	0.356
50	0.000	0.348	0.000	0.511	0.000	0.511	0.000	0.511
51	0.000	0.141	0.000	0.200	0.000	0.200	0.000	0.200
52	0.000	0.265	0.000	0.467	0.000	0.467	0.000	0.467
53	0.000	0.141	0.000	0.200	0.000	0.200	0.000	0.200
54	0.000	0.141	0.000	0.200	0.000	0.200	0.000	0.200
55	0.000	0.141	0.000	0.200	0.000	0.200	0.000	0.200
56	0.000	0.000	0.000	0.000	0.000	0.000	0.000	0.000
57	0.000	0.348	0.000	0.511	0.000	0.511	0.000	0.511
58	0.000	0.000	0.000	0.000	0.000	0.000	0.000	0.000
59	0.000	0.000	0.000	0.000	0.000	0.000	0.000	0.000
60	0.000	0.091	0.000	0.059	0.000	0.000	0.000	0.100
61	0.000	0.413	0.000	0.622	0.000	0.622	0.000	0.622
62	0.000	0.217	0.000	0.356	0.000	0.356	0.000	0.356
63	0.000	0.292	0.000	0.533	0.000	0.533	0.000	0.533
64	0.000	0.301	0.000	0.556	0.000	0.556	0.000	0.556
65	0.000	0.000	0.000	0.000	0.000	0.000	0.000	0.000
66	0.000	0.000	0.000	0.000	0.000	0.000	0.000	0.000
67	0.000	0.141	0.000	0.200	0.000	0.200	0.000	0.200
68	0.000	0.348	0.000	0.511	0.000	0.511	0.000	0.511
69	0.000	0.217	0.000	0.356	0.000	0.356	0.000	0.356
70	0.000	0.141	0.000	0.200	0.000	0.200	0.000	0.200
71	0.000	0.447	0.000	0.689	0.000	0.689	0.000	0.689
72	0.000	0.217	0.000	0.356	0.000	0.356	0.000	0.356
73	0.000	0.217	0.000	0.356	0.000	0.356	0.000	0.356
74	0.000	0.217	0.000	0.356	0.000	0.356	0.000	0.356
75	0.000	0.217	0.000	0.356	0.000	0.356	0.000	0.356
76	0.000	0.278	0.000	0.378	0.000	0.378	0.000	0.378
77	0.000	0.410	0.000	0.644	0.000	0.644	0.000	0.644
78	0.000	0.473	0.000	0.644	0.000	0.644	0.000	0.644
79	0.000	0.000	0.000	0.000	0.000	0.000	0.000	0.000
80	0.000	0.348	0.000	0.511	0.000	0.511	0.000	0.511
81	0.000	0.265	0.000	0.467	0.000	0.467	0.000	0.467
82	0.000	0.292	0.000	0.533	0.000	0.533	0.000	0.533
83	0.000	0.292	0.000	0.533	0.000	0.533	0.000	0.533
84	0.000	0.141	0.000	0.200	0.000	0.200	0.000	0.200
85	0.000	0.000	0.000	0.000	0.000	0.000	0.000	0.000
86	0.000	0.000	0.000	0.000	0.000	0.000	0.000	0.000
87	0.000	0.000	0.000	0.000	0.000	0.000	0.000	0.000
88	0.000	0.265	0.000	0.467	0.000	0.467	0.000	0.467
89	0.000	0.217	0.000	0.356	0.000	0.356	0.000	0.356
90	0.000	0.390	0.000	0.600	0.000	0.600	0.000	0.600
91	0.000	0.000	0.000	0.000	0.000	0.000	0.000	0.000
92	0.000	0.000	0.000	0.000	0.000	0.000	0.000	0.000
93	0.000	0.141	0.000	0.200	0.000	0.200	0.000	0.200
94	0.000	0.292	0.000	0.533	0.000	0.533	0.000	0.533
95	0.000	0.217	0.000	0.356	0.000	0.356	0.000	0.356
96	0.000	0.292	0.000	0.533	0.000	0.533	0.000	0.533
97	0.000	0.000	0.000	0.000	0.000	0.000	0.000	0.000
98	0.000	0.000	0.000	0.000	0.000	0.000	0.000	0.000
99	0.000	0.141	0.000	0.200	0.000	0.200	0.000	0.200
100	0.000	0.000	0.000	0.000	0.000	0.000	0.000	0.000
101	0.000	0.413	0.000	0.622	0.000	0.622	0.000	0.622
102	0.000	0.301	0.000	0.556	0.000	0.556	0.000	0.556
103	0.000	0.265	0.000	0.467	0.000	0.467	0.000	0.467
104	0.000	0.141	0.000	0.200	0.000	0.200	0.000	0.200

ID	1 - SN (train)	1 - SN (test)	1 - SO (train)	1 - SO (test)	1 - SS (train)	1 - SS (test)	1 - SZ (train)	1 - SZ (test)
105	0.000	0.141	0.000	0.200	0.000	0.200	0.000	0.200
106	0.000	0.413	0.000	0.622	0.000	0.622	0.000	0.622
107	0.000	0.000	0.000	0.000	0.000	0.000	0.000	0.000
108	0.000	0.000	0.000	0.000	0.000	0.000	0.000	0.000
109	0.000	0.000	0.000	0.000	0.000	0.000	0.000	0.000
110	0.000	0.000	0.000	0.000	0.000	0.000	0.000	0.000
111	0.000	0.000	0.000	0.000	0.000	0.000	0.000	0.000
112	0.000	0.000	0.000	0.000	0.000	0.000	0.000	0.000
113	0.001	0.001	0.002	0.002	0.185	0.166	0.004	0.004
114	0.239	0.233	0.392	0.386	0.607	0.618	0.506	0.504
115	0.171	0.262	0.272	0.385	0.272	0.382	0.350	0.481
116	0.200	0.231	0.285	0.354	0.280	0.347	0.347	0.435

Table H: Stability values for the Pareto optimal configurations in Table E. The ID column references Table E. "—" means that the configuration is not Pareto optimal for the corresponding stability measure.

Stomach

ID	Classifier	Parameters	Filter	`n.feats`	Error (train)	Error (test)	Size (train)	Size (test)
117	Lasso. Log. Reg.	$\lambda = 0.0001$	AUC	2730	0.215	0.466	2726.9	2726.1
118	Lasso. Log. Reg.	$\lambda = 0.00004$	MRMR	1972	0.219	0.491	1971.1	1970.2
119	GLM Boosting	$m_{\text{stop}} = 153$	MRMR	461	0.193	0.360	35.5	46.8
120	GLM Boosting	$m_{\text{stop}} = 17$	AUC	8330	0.221	0.494	11.8	14.0
121	GLM Boosting	$m_{\text{stop}} = 228$	Variance	8526	0.175	0.461	48.7	60.9

Table I: All Pareto optimal configurations for data set Stomach (above the horizontal line) and accuracy optimal configurations (below the horizontal line). The ID column references Tables J, K, and L.

ID	1 - SJ (train)	1 - SJ (test)	1 - SC (train)	1 - SC (test)	1 - SD-1 (train)	1 - SD-1 (test)	1 - SD-10 (train)	1 - SD-10 (test)
117	0.352	0.441	—	—	—	—	—	—
118	0.368	0.457	0.281	0.369	0.609	0.694	—	—
119	0.594	0.747	0.426	0.601	0.656	0.749	—	—
120	0.596	0.864	0.428	0.767	0.673	0.815	0.684	0.827
121	0.664	0.739	0.502	0.591	0.708	0.739	0.752	0.794

Table J: Stability values for the Pareto optimal configurations in Table I. The ID column references Table I. "—" means that the configuration is not Pareto optimal for the corresponding stability measure.

ID	1 - SD-0 (train)	1 - SD-0 (test)	1 - SD-2 (train)	1 - SD-2 (test)	1 - SD (train)	1 - SD (test)	1 - SL (train)	1 - SL (test)
117	0.401	0.483	—	—	0.215	0.284	—	—
118	0.412	0.497	—	—	0.226	0.296	—	—
119	0.652	0.744	0.659	0.754	0.425	0.599	—	—
120	0.672	0.813	0.675	0.816	0.430	0.766	0.379	0.755
121	0.703	0.733	0.713	0.745	0.500	0.588	0.482	0.577

Table K: Stability values for the Pareto optimal configurations in Table I. The ID column references Table I. "—" means that the configuration is not Pareto optimal for the corresponding stability measure.

ID	1 - SN (train)	1 - SN (test)	1 - SO (train)	1 - SO (test)	1 - SS (train)	1 - SS (test)	1 - SZ (train)	1 - SZ (test)
117	0.135	0.183	0.215	0.284	—	—	0.313	0.398
118	0.142	0.192	0.226	0.296	0.253	0.333	0.335	0.419
119	0.307	0.451	0.424	0.599	0.421	0.597	—	—
120	0.320	0.624	0.427	0.766	0.423	0.768	0.532	0.789
121	0.372	0.437	0.499	0.587	0.498	0.587	0.605	0.689

Table L: Stability values for the Pareto optimal configurations in Table I. The ID column references Table I. "—" means that the configuration is not Pareto optimal for the corresponding stability measure.
